# Intramedullary nailing has sufficient durability for metastatic femoral fractures

**DOI:** 10.1186/s12957-016-0836-2

**Published:** 2016-03-10

**Authors:** Takaaki Tanaka, Jungo Imanishi, Chris Charoenlap, Peter F. M. Choong

**Affiliations:** Department of Orthopaedics, St. Vincent’s Hospital Melbourne, 41 Victoria Parade, Fitzroy, Victoria 3065 Australia; Department of Orthopaedic Surgery, Osaka University Graduate School of Medicine, 2-2 Yamadaoka, Suita, Osaka Japan; Department of Surgery, St. Vincent’s Hospital Melbourne, Level 2, Clinical Sciences Building, 29 Regent Street, Fitzroy, 3065 Victoria Australia; Bone and Soft Tissue Sarcoma Unit, Peter MacCallum Cancer Centre, 2 St Andrews Place, East Melbourne, 3002 Victoria Australia

**Keywords:** Metastatic femoral fracture, Intramedullary nailing, Postoperative survival, Implant survival

## Abstract

**Background:**

Surgical treatment options of femoral metastases include intramedullary nailing (IMN) and endoprosthetic reconstruction (EPR). Previous studies have demonstrated functional and oncological advantages of EPR over IMN. The purpose of this study was to (1) report the durability of IMN and (2) establish the indication of IMN for femoral metastases.

**Methods:**

In 2003–2013, among 186 surgically treated femoral metastasis cases, we retrospectively reviewed 80 consecutive IMN cases in 75 patients, including 14 pathological and 66 impending fractures. For the decision of surgical procedure (IMN, EPR, or plating), the following factors are considered: (1) fracture pattern (impending or pathological fracture), (2) Mirels’ score (≥8 or <8), (3) fracture site (femoral head, neck, intertrochanter, subtrochanter, diaphysis, or distal), (4) number of metastases (solitary or multiple), and (5) patient’s estimated prognosis. Patient demographics, postoperative survival, implant survival, and early postoperative mortality were reviewed.

**Results:**

The patients were 37 males and 38 females, with a mean age of 60.1 (20–84) years. Average follow-up period was 11.4 (1–77) months. The most common fracture site was the subtrochanter (46/80), followed by the diaphysis (26/80) and the intertrochanter (8/80). The most common primary tumor was lung cancer (24/80, 32 %), followed by breast cancer (24 %) and melanoma (15 %). With the exception of six cases, all patients underwent postoperative radiotherapy to the affected whole femur. The postoperative patient survival was 14.2 and 8.4 % at 2 and 3 years from surgery, respectively, while the implant survival rate remained 94.0 % at both 2 and 3 years. Three out of 46 subtrochanteric cases required revision surgeries because of proximal breakage of implant 4–50 months after initial surgery for femoral metastases, but all were replaced by mega-prosthesis and did not need further operation until their death. Early postoperative fatal complications were observed in three patients, all of which were pulmonary dysfunction.

**Conclusions:**

The performance of IMN in this study was satisfactory although a large portion of sub- and intertrochanter metastases were included. Broader indication including these parts should be considered, for IMN has advantages such as lower cost and less invasiveness and even an implant failure can be revised by mega-prosthetic reconstruction.

## Background

The femur is one of the most common sites for bone metastases [1, 2]. Metastatic femoral fracture affects not only a patient’s prognosis but also their quality of life and ambulation [3–5]. Surgical procedures for femoral metastases are widely chosen from intramedullary nailing (IMN) [6–16], endoprosthetic reconstruction (EPR) [7–14], or plating and cementation [7, 8, 11, 12], but how to choose the best treatment for each case is still uncertain, especially for inter- and subtrochanteric metastases [8, 9, 14, 15].

Some previous studies demonstrated that the rate of implant failure in the EPR group was lower than that in the IMN group, and overall patient survival was also longer for the EPR group than the IMN group [9–11]. However, it is impossible to remove any treatment bias related to the patient’s general condition and primary tumor before the procedure for both groups in such studies. By contrast, IMN has some advantages over EPR, including lower cost and less invasiveness. Considering these benefits, if IMN is durable throughout the expected lifespan for patients with metastases, IMN can become a primary surgical option for patients affected by femoral metastases.

The aim of this study was to (1) accurately measure the implant survival of IMN at our institution and (2) reconsider the indication of IMN for femoral metastatic lesions.

## Methods

**Fig. 1:**
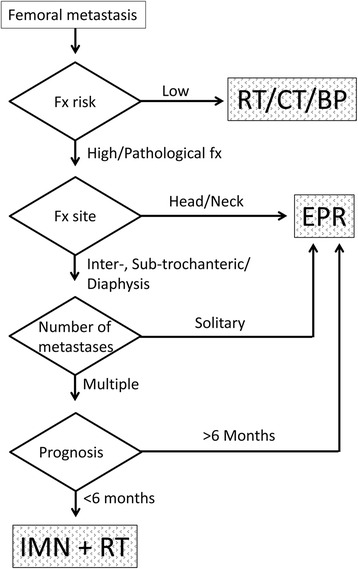
Flowchart of our institutional strategy for procedural selection. For the decision of surgical procedure (IMN or EPR), the following factors are considered: (1) fracture pattern (impending or pathological fracture), (2) Mirels’ score (≥8 or <8), (3) fracture site (femoral head, neck, intertrochanter, subtrochanter, diaphysis, or distal), (4) number of metastases (solitary or multiple), and (5) patient’s estimated prognosis. *IMN* intramedullary nailing, *EPR* endoprosthetic reconstruction, *Fx* fracture, *RT* radiotherapy, *CT* chemotherapy, *BP* bisphosphonate

In our institution, before the decision of surgery and procedure type (IMN, EPR, or plating), several factors are considered, (1) fracture pattern (impending or pathological fracture), (2) fracture risk (Mirels’ score of ≥8 or <8 [17]) for impending fracture, (3) fracture site (femoral head, neck, intertrochanter, subtrochanter, diaphysis, or distal), (4) number of metastases (solitary or multiple), (5) patient’s estimated prognosis (≥6 or <6 months), and (6) patient’s preference after informed consent. A flowchart in Fig. 1 demonstrates our strategy concerning the procedural selection. IMN was performed with either the Trigen System (Smith & Nephew, Memphis, TN, USA) or the Alta CFx IM rod system (Howmedica, Rutherford, NJ, USA). Among IMN procedures, the patients routinely underwent postoperative radiotherapy to the affected whole femur at approximately 2 weeks after the surgery.

According to the database at our institution, 186 surgeries for femoral metastases with pathological or impending fracture were identified over a 10-year period, from June 2003 to June 2013. Among the 186 cases, there were 95 EPR, 80 IMN, 8 plating and screw with cementation, and 3 Girdlestone procedures. The 80 consecutive IMN procedures in 75 patients (37 males and 38 females) were retrospectively reviewed in terms of patient demographic data, postoperative survival, implant survival, and early fatal postoperative complications. Postoperative survival was calculated from the date of administering IMN procedure to the date of death or last follow-up. Implant survival was defined from the date of administering IMN procedure to the date of implant failure, death, or last follow-up. The average age and follow-up period were 60.1 years (20–80 years) and 11.4 months (1–77 months), respectively. Impending fractures accounted for 82.5 % (66 of 80) and pathological fractures made up 17.5 % (14 of 80). The majority of the cases had multiple metastases (74 of 80, 92.5 %). Ten percent of the lesions (8 of 80) occurred in the intertrochanteric area, 57.5 % (46 of 80) in the subtrochanteric area, and 32.5 % (26 of 80) in the shaft of femur. The most common primary tumor was lung cancer (32.0 %, 24 of 80), followed by breast cancer (24.0 %, 18 of 80) (Table 1).

**Table 1:** Patient characteristics for IMN

Characteristics	IMN
Number of patients	75 (37 males/38 females)
Number of cases	80 (38 males/42 females)
Average ages (year)	60.1 (range, 20–80)
Average follow up (months)	11.4 (range, 1–77)
Fracture pattern	
Impending	66 (82.5 %)
Survival (<6 months/>6 months)	35 (53 %)/31 (47 %)
Pathological	14 (17.5 %)
Survival (<6 months/>6 months)	6 (42.9 %)/8 (57.1 %)
Number of metastases	
Solitary	6 (7.5 %)
Survival (<6 months/>6 months)	1 (16.7 %)/5 (83.3 %)
Multiple	74 (92.5 %)
Survival (<6 months/>6 months)	40 (54.1 %)/34 (45.9 %)
Site of metastasis	
Head/neck	0 (0 %)
Intertrochanteric	8 (10 %)
Subtrochanteric	46 (57.5 %)
Diaphysis	26 (32.5 %)
Primary tumor	
Lung	24 (32.0 %)
Breast	18 (24.0 %)
Melanoma	11 (14.7 %)
Renal	5 (6.7 %)
Prostate	5 (6.7 %)
Unknown	4 (5.3 %)
Others	8 (10.6 %)
Radiotherapy	
Yes	74 (92.5 %)
No	6 (7.5 %)

*IMN* intramedullary nailing

Kaplan-Meier survival curves using postoperative survival and implant survival were calculated using IBM SPSS version 17.0 (SPSS Inc., Chicago, IL, USA). The event for postoperative survival and implant survival is death and implant failure, respectively.

## Results and discussion

The patient background is summarized in Table 1. Eighty IMN procedures were performed in 75 patients, 37 males and 38 females. Five patients underwent bilateral IMN in separate procedures at least 8 days apart. Seventy-four cases underwent radiotherapy after IMN procedure; however, six out of 80 cases were unable to undergo radiotherapy due to postoperative poor medical problems (Table 1).

**Fig. 2:**
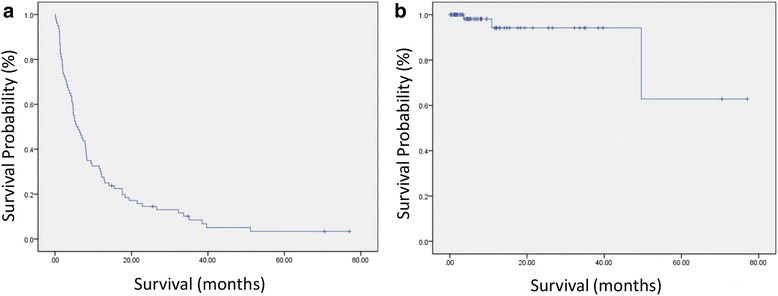
Survival curve for postoperative and implant survival. Kaplan-Meier survival analysis shows for postoperative survival (**a**) and implant survival (**b**)

The 2- and 3-year postoperative survivals were 14.2 and 8.4 %, respectively (Fig. 2a). In contrast, the implant survival rate was 94.0 % at both 2 and 3 years; however, it dropped to 62.8 % at 50 months (Fig. 2b).

**Fig. 3:**
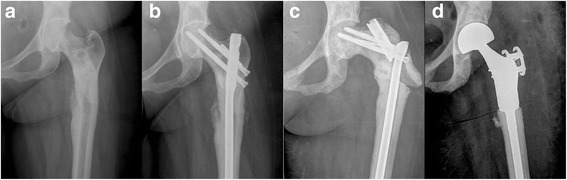
The clinical course of one patient who undergone revision surgery after implant failure. X-ray imaging revealed an impending fracture and an osteoblastic lesion in the left subtrochanteric part of femur (**a**). IMN was performed, and the postoperative course was uneventful until the implant failure at the 50-month clinical follow-up (**b**). X-ray imaging showed a broken implant in proximal part (**c**). The IM rod was removed and the proximal part of the femur was resected and then replaced with EPR (**d**)

In this study, three intramedullary nails broke through their proximal parts (Table 2). For all three patients, the fracture site was subtrochanteric and the implant failures showed a similar pattern of breakage at the proximal part of the IM rod. The IM rods were removed in all three cases, and the proximal part of the femurs were resected and then replaced with EPRs. The three patients did not require any further operations until death. Figure 3 shows the clinical course of case 2 patient who was a 49-year female and had a 5-year history of breast cancer, who presented with severe left femoral pain, and the patient Mirels’ score was 10. The patient underwent IMN procedure for impending fracture. Fifty months after the first surgery, she underwent EPR as a revision surgery due to implant breakage. The patient did not require any further operations until she died after 1.5 months. Table 3 shows three early postoperative deaths on postoperative days 3, 7, and 12. Two of the three patients (cases 1 and 3) underwent palliative therapy before the surgery, but complained of severe femoral pain with Mirels’ score of 10. One patient (case 2) was transferred to the emergency department due to pathological fracture. All the patients had several comorbidities including lung, liver, adrenal, brain, and multiple bone metastases, and the cause of death in all three cases were respiratory failure (Table 3).

**Table 2:** IMN implant failure cases

Case No.	Age, sex	Primary cancer	Fx pattern	Fx site	Complication	Time to failure (months)	Treatment
1	58, F	Breast	Pathological	Subtrochanteric	Nonunion and nail breakage	4	Revision with EPR
							No further complication
2	49, F	Breast	Impending	Subtrochanteric	Nail breakage	50	Revision with EPR
							No further complication
3	20, F	Pheochromocytoma	Impending	Subtrochanteric	Nail breakage	11	Revision with EPR
							No further complication

*IMN* intramedullary nailing, *M* male, *F* female, *Fx* fracture, *EPR* endoprosthetic reconstruction

**Table 3:** Postoperative fatal complications within 14 days

Case No.	Age, sex	Primary cancer	Fx pattern	Fx site	Time to death (days)	Treatment
1	78, M	Melanoma	Impending	Proximal shaft	7	Palliative therapy
						Multiple metastases: lung, liver, adrenal, and bones
						Mirels’ score 10, severe femoral pain
						Cause of death: respiratory complication
2	69, M	Prostate	Pathological	Subtrochanteric	3	Multiple metastases: lung and bones
						Pathological fracture managed with IMN insertion
						Cause of death: heart dysfunction and respiratory complication
3	48, F	Lung	Impending	Proximal shaft	12	Palliative therapy
						Multiple metastases: brain and bones
						Mirels score 10, severe femoral pain
						Cause of death: respiratory complications

*M* male, *F* female, *Fx* fracture, *IMN* intramedullary nailing

The goal of surgical treatment of femoral metastatic fractures is not only to internally fix or prevent pathological fracture but also to reduce pain and optimize recovery, mobility, or care for the patient with minimal invasiveness and complications [3–5, 14]. The strategy is to ensure the durability of treatment, and in this regard, implant survival should exceed patient survival after surgery.

Our study has several potential limitations. Firstly, this study is a single-center retrospective study with all the limitations inherent to such design. Secondly, there were some metastatic femoral fracture cases that were not treated according the indication pathway because of surgeon and patient preferences. Thirdly, patients with metastases underwent not only surgery but also adjuvant therapies, such as chemotherapy, radiotherapy, and hormonal therapy. Postoperative survival may be affected by those therapies. However, in this study, there were 186 consecutive procedures for metastatic femoral fractures, and 80 IMN cases, suggesting a degree of external validity and robustness.

Surgical treatment strategy towards femoral metastases still remains unclear. Both IMN and EPR for femoral metastases are widely performed. IMN has some advantages over EPR, such as lower cost, less blood loss, less muscle wasting, shorter operation time, and shorter hospitalization. On the other hand, some studies have reported that EPR is associated with higher patient survival [9], lower mechanical failure rate, and more durability than IMN [10, 11]. However, those studies did not consider the differences in patient demographics, and thus comparing outcomes between IMN and EPR in these reports may not be appropriate.

Treatment indications vary between institutions. Table 4 shows peer-reviewed articles from 2008 through 2013 and our current study, describing IMN, EPR, plating and cementation, and other surgical procedures in femoral metastases [7–13]. These studies can be divided into two groups. Among four studies, the proportion of IMN/EPR is approximately 1:2 [7, 10–12]. Nilsson at al. reported that they did not perform IMN for trochanteric or subtrochanteric metastases [7]. Steensma et al. and Alvi at el. avoided IMN for the patients with intertrochanteric lesions of the femur [11, 12]. In the other group, the proportions of IMN and EPR were nearly equal [8, 9, 13]. Sarahrudi et al. reported that their IMN group included inter- and subtrochanteric metastases [8], and Mavrogenis et al. reported also performing IMN for fractures in the intertrochanteric part of the femur except for metastases invading the articular surface [9]. These reports included a broader indication for IMN. Regarding the superiority of EPR to IMN and vice versa, while Mavrogenis at al. reported a significantly higher survival in patients with EPR [9], Sarahrudi et al. reported that EPR and IMN were equivalent in terms of safety [8]. A few other articles have also mentioned good outcomes with IMN [15, 16].

**Table 4:** Previous reports regarding surgical procedures for femoral metastatic lesions

Study	Number of case	IMN	EPR	Plating and cementation	Other procedure	Result (reoperation, complication)
Nilsson at al. [7] 2008	245	55 (22.4 %)	157 (64.1 %)	30 (12.2 %)	3 (1.2 %)	Reoperation: 1.8 % IMN, 9.1 % ORIF
Sarahrudi et al. [8] 2009	139	94 (67.6 %)	23 (16.5 %)	15 (10.8 %)	7 (5.1 %)	Complication: 3.2 % IMN, 8.6 % EPR, 20 % ORIF
Mavrogenis et al. [9] 2011	110	53 (48.2 %)	57 (51.8 %)	–	–	Complication: 1.9 % IMN, 8.8 % EPR
Weiss et al. [13] 2013	196	108 (55.1 %)	82 (41.8 %)	–	6 (3.1 %)	Reoperation: 9.3 % IMN, 6.1 % ORIF
Harvey at al. [10] 2012	159	46 (28.9 %)	113 (71.1 %)	–	–	Reoperation: 26.1 % IMN, 8.0 % EPR
						Revision: 21.7 % IMN, 2.7 % EPR
Steensma at al. [11] 2012	298	82 (27.5 %)	197 (66.1 %)	19 (6.4 %)	–	Reoperation: 6.1 % IMN, 3.0 % EPR, 42.1 % ORIF
Alvi at al. [12] 2013	53	16 (30.2 %)	36 (67.9 %)	1 (1.9 %)	–	Revision: 35.6 % IMN
Current study	186	80 (43.0 %)	95 (51.1 %)	8 (4.3 %)	3 (1.6 %)	Revision: 3.8 % IMN

*IMN* intramedullary nailing, *EPR* endoprosthetic reconstruction, *ORIF* open reduction and internal fixation

As far as we know, there is no strong evidence concerning the indication of IMN for subtrochanteric metastases. The evidence grade of EPR for femoral neck fracture is grade B which indicates consistent, fair (level II or III) evidence, while IMN is grade C which indicates conflicting or poor-quality (level IV or V) evidence, including inter- and subtrochanteric fractures, except for intertrochanteric impending fracture whose grade is B [14]. In our institution, the postoperative survival was 14.2 and 8.4 % at 2 and 3 years, respectively, and the implant survival rate was 94.0 % at both 2 and 3 years. Harvey et al. noted that the IMN implant survival rate was 85 % at 2 years [10], and Steensma et al. noted that the IMN implant survival rate was 88 % at 3 years [11]. Compared to these reports, our IMN implant survival rate of 94 % at 3 years is comparable and can be regarded as appropriate for a patient population whose survival at 3 years is only 8.4 %.

There were three patients who underwent revisions due to implant breakage. In all such cases, the site was in the subtrochanteric part of the femur, and failure occurred in the proximal part of the nail. EPR was chosen as the revision procedure in order to prevent further complications after revision. Forsberg et al. recommended the use of EPR as a salvage procedure even at the end of life [3], and we concur with their recommendation.

In our study, three cases had early fatal complications after surgery (3.8 %), all of which had lung metastases at the time of IMN procedure. The role of IMN should be carefully considered in patients who have pre-existing pulmonary dysfunction. Moon et al. reported that prophylactic IMN did not appear to be safer than curative IMN for femoral fractures [18]. Barwood et al. reported that acute oxygen desaturation and hypotension occurred in 24.4 % of patients during IMN procedures for metastatic femoral fractures and 6.6 % of patients died from cardiorespiratory dysfunctions during the perioperative period [19].

Cost-effective treatment for bone metastases is important because of the already high cost of treating malignancy [20]. Schulman et al. noted that total medical cost for patients with bone metastases was significantly higher than that for patients without bone metastases [21]. A cost-effectiveness analysis between IMN and EPR should be studied further.

## Conclusions

In this report, the performance of IMN with much broader indication including the trochanteric part of the femur is sufficient for a few years. IMN has several advantages for patients with femoral metastatic fractures, such as lower cost, less invasiveness, wider indication, sufficient durability, and revision options. Therefore, other than EPR, IMN is a suitable procedure for patients with femoral metastatic fracture even in trochanteric part.

## Ethics approval and consent to participate

This study was conducted at St. Vincent’s Hospital Melbourne, in accordance with the World Medical Association Declaration of Helsinki. The research protocol was approved by the institutional human research ethics committee (HREC number: QA109/14), and waived off the requirement for informed consent from the subjects. We declare that we have no conflicts of interest.

## Abbreviations

IMN: Intramedullary nailing
EPR: Endoprosthetic reconstruction
